# ‘We have goals but [it is difficult]’. Barriers to antiretroviral therapy adherence among women using alcohol and other drugs living with HIV in South Africa

**DOI:** 10.1111/hex.13422

**Published:** 2022-01-21

**Authors:** Jacqueline W. Ndirangu, Margaret W. Gichane, Felicia A. Browne, Courtney P. Bonner, William A. Zule, Erin N. Cox, Kevin M. Smith, Tara Carney, Wendee M. Wechsberg

**Affiliations:** ^1^ Substance Use, Gender, and Applied Research Program, RTI International Research Triangle Park North Carolina USA; ^2^ Gillings School of Global Public Health University of North Carolina at Chapel Hill Chapel Hill North Carolina USA; ^3^ Alcohol, Tobacco and Other Drug Research Unit South African Medical Research Council Tygerberg South Africa; ^4^ Department of Psychiatry and Mental Health University of Cape Town Rondebosch Cape Town South Africa; ^5^ Department of Psychology North Carolina State University Raleigh North Carolina USA; ^6^ Psychiatry and Behavioral Sciences Duke University School of Medicine Durham North Carolina USA

**Keywords:** alcohol and other drugs (AOD), antiretroviral therapy (ART), gender‐focused, HIV care continuum, implementation science, socioecological barriers

## Abstract

**Background:**

Women living with HIV who misuse alcohol and live in economically disadvantaged settings in South Africa experience a multitude of contextual barriers as they navigate the HIV care continuum. The Women's Health CoOp (WHC), a brief, woman‐focused, behavioural, evidence‐based intervention, has been shown to be effective in reducing heavy drinking and improving HIV‐related outcomes among this key population. However, these women face other broader socioecological barriers to antiretroviral therapy (ART) adherence.

**Methods:**

The WHC was implemented in a modified, stepped‐wedge implementation science trial in public health clinics and substance use treatment programmes in Cape Town, South Africa. A qualitative substudy was conducted to explore barriers to HIV treatment adherence among women enrolled in this trial. Eight focus group discussions were conducted with 69 participants 6 months after completion of the WHC workshops. Focus groups were audio‐recorded (with consent), transcribed verbatim and analysed using a thematic approach.

**Results:**

The mean age of the participants was 33 years and the mean self‐reported number of drinks per day was 13. The main contextual factors influencing participants’ ART adherence were intrapersonal‐level factors (substance use, financial constraints, food insecurity; community‐level factors (anticipated and enacted stigma, community violence) and institutional‐level factors (patient–provider relationships, health facility barriers, environmental stigma).

**Conclusion:**

Comprehensive interventions addressing the contextual barriers and unique challenges faced by women who misuse alcohol in low‐resource settings that intersect with HIV treatment nonadherence should be implemented in tandem with successful biobehavioural HIV interventions for long‐term effectiveness and sustainability.

**Patient or Public Contribution:**

Our South African community collaborative board has been involved throughout this study; participants and clinic staff voices have been essential in our interpretation of these findings.

## 1 INTRODUCTION

In 2016, South Africa adopted the World Health Organization's Universal Test and Treat policy, making all people living with HIV eligible for antiretroviral therapy (ART) at diagnosis.^1^ However, despite successful scale‐up of HIV testing and treatment, suboptimal retention in care and poor viral suppression through nonadherence to ART continues to be a major challenge.^1^ Women aged 25–49, experience a disproportionate burden of HIV prevalence (33.3%) as compared with men (19.4%); however, only 69%–75% of women on ART are virally suppressed, which is short of the UNAIDS 95‐95‐95 targets by 2030.^2^, ^3^

Various intrapersonal‐level factors have been identified to help understand the causes of HIV treatment nonadherence and barriers that women living with HIV face as they try to remain in HIV care. One barrier of concern is substance use, which has been associated with reduced HIV adherence and HIV disease progression.^4^, ^5^, ^6^ Estimates suggest that in 2012, approximately 2900 HIV‐related deaths and 11,400 years lived with a disability among women living with HIV in South Africa were attributable to substance use and its effect on nonadherence to ART.^7^ Other contextual barriers to ART adherence among women living with HIV include gender‐based violence and a history of social, legal, and economic disempowerment and gender inequality that impact women's ability to engage in care.^8^, ^9^, ^10^, ^11^ Consequently, the intersecting syndemic of HIV, substance use and gender inequality necessitates multifaceted interventions to mitigate the risk of nonadherence and poor health outcomes among women on the individual level.^10^, ^12^, ^13^

The Women's Health CoOp (WHC), a brief, woman‐focused, behavioural, evidence‐based intervention, grounded in empowerment and feminist theory is one such intervention. The WHC uses a skill‐building approach to reduce varying risk behaviours, including alcohol and other drug (AOD) use, sexual risk behaviour, gender‐based violence and biomedical knowledge of HIV and sexually transmitted infections (STIs). Based on an intervention developed in the US for African American women who use substances and adapted to various key populations and settings, the WHC has been found to be efficacious in reducing HIV risk for women who use AODs.^9^, ^14^, ^15^ In a recent cluster‐randomized trial of the WHC with biobehavioural approaches, women in the WHC arm had greater reductions in heavy drinking and other risk‐related outcomes, and greater reductions in HIV viral load were observed for a subsample of WHC participants living with HIV.^14^

However, addressing the nexus of substance use, gender inequality and HIV is only part of a larger socioecological framework. Other factors that converge with substance use, such as food insecurity and financial constraints, have been found to be strong predictors of suboptimal HIV treatment outcomes.^16^, ^17^, ^18^ Additionally, stigma, access to healthcare and institutional and health‐system factors may undermine the sustainability of successful behaviour change evidence‐based interventions focused on ART adherence.^19^, ^20^, ^21^ The present study explored how multiple factors of the socioecological framework impact HIV treatment and adherence among women living with HIV who use AODs.

## 2 METHODS

This qualitative study was part of a larger implementation science research study to evaluate the effectiveness of the WHC for women living with HIV and who reported AOD use in Cape Town, South Africa.^10^, ^22^ The WHC intervention included two interactive group workshops that combined risk‐reduction information about AODs, ART initiation, understanding the importance of ART adherence and STIs. Workshops also included material on behavioural skills training, such as practicing male and female condom use, and reducing sexual risk through negotiation and communication skills. The WHC was implemented in four public healthcare clinics with HIV/antenatal clinics and four substance use treatment clinics (hereafter known as Matrix programs), all located in economically underserved communities in Cape Town, from 2015 to 2018 using a modified stepped‐wedge design of four cycles.^10^, ^22^ Each implementation cycle lasted 6 months. Focus group discussions were conducted with study participants approximately 6 months after women had attended the WHC workshops to assess the barriers to ART adherence from a socioecological framework.

### 2.1 Recruitment and data collection

The 480 women who participated in the WHC implementation science trial met the following eligibility criteria: (1) being between the ages of 18 and 45 years; (2) self‐reporting the use of at least one drug, which could include alcohol, at least weekly during the previous 3 months; (3) reporting unprotected sex (sex without a condom) with a male partner in the past 6 months; (4) having a positive verifiable HIV test result; (5) reporting the intention to remain in the study area for at least the next 6 months; (6) providing contact information and (7) being willing to participate in AOD use screening.^10^ An additional criterion for participation in the focus group discussions was the completion of both WHC intervention workshops, which 84% of trial participants met. Eligible participants from each of the eight study clinics in which they were enrolled were randomly within each cycle selected and contacted for the follow‐up focus group discussions. A total of 110 women were contacted from across the cycles, with 69 attending the focus group discussion. We conducted eight focus group discussions—one focus group discussion from each of the eight clinics consisting of between 5 and 11 participants in each group (median = 9). Each focus group lasted approximately 1 hour.

All focus group discussions were conducted in a private room at the research study site. Participants provided written and signed informed consent before each focus group. Focus group discussions were primarily conducted in English by trained research multilingual project staff, some of whom acted as real‐time interpreters when translations in isiXhosa and Afrikaans, two of the primary languages spoken in Cape Town, were needed. These staff were trained in understanding the focus group questions and the ability to translate the meaning of participants’ answers and comments as discussion happened. Focus group discussion guides were semistructured, consisting of open‐ended questions and prompts to explore multiple topics, including participants’ feedback on the WHC workshops and behavioural and contextual factors that influenced their ART adherence, such as barriers experienced as participants tried to navigate through HIV treatment.

### 2.2 Analysis

All focus group discussions were audio‐recorded (with consent) and transcribed by trained research staff. A second staff transcriber reviewed recordings and transcripts to ensure completeness. We used an applied thematic analysis approach to guide analysis,^23^ we began by conducting a deep reading of the transcripts to familiarize ourselves with the data and wrote memos to identify recurring concepts. We developed an initial codebook using a priori codes based on the Focus Group Discussion guide and common concepts observed during transcript review. Interrater reliability was assessed using Cohen's kappa *(*κ).^24^ One analyst applied codes to the transcripts and the other analyst coded the same transcript blinded to the first analyst's codes. Analysts then met several times to compare coding, refine code definitions and resolve disagreements. On reaching a high agreement (κ = 0.81), the remaining transcripts were double coded by the two analysts. Coded data were summarized in visual matrices to identify themes within and across focus group discussions. Dedoose software (v.8.0.42) was used for the management, coding and analysis of the data.

### 2.3 Ethics

This study protocol was approved by the South African Medical Association Research Ethics Committee (SAMAREC); City of Cape Town: City Health Research Committee, and the RTI International Committee for the Protection of Human Subjects.

## 3 RESULTS

**Figure 1:**
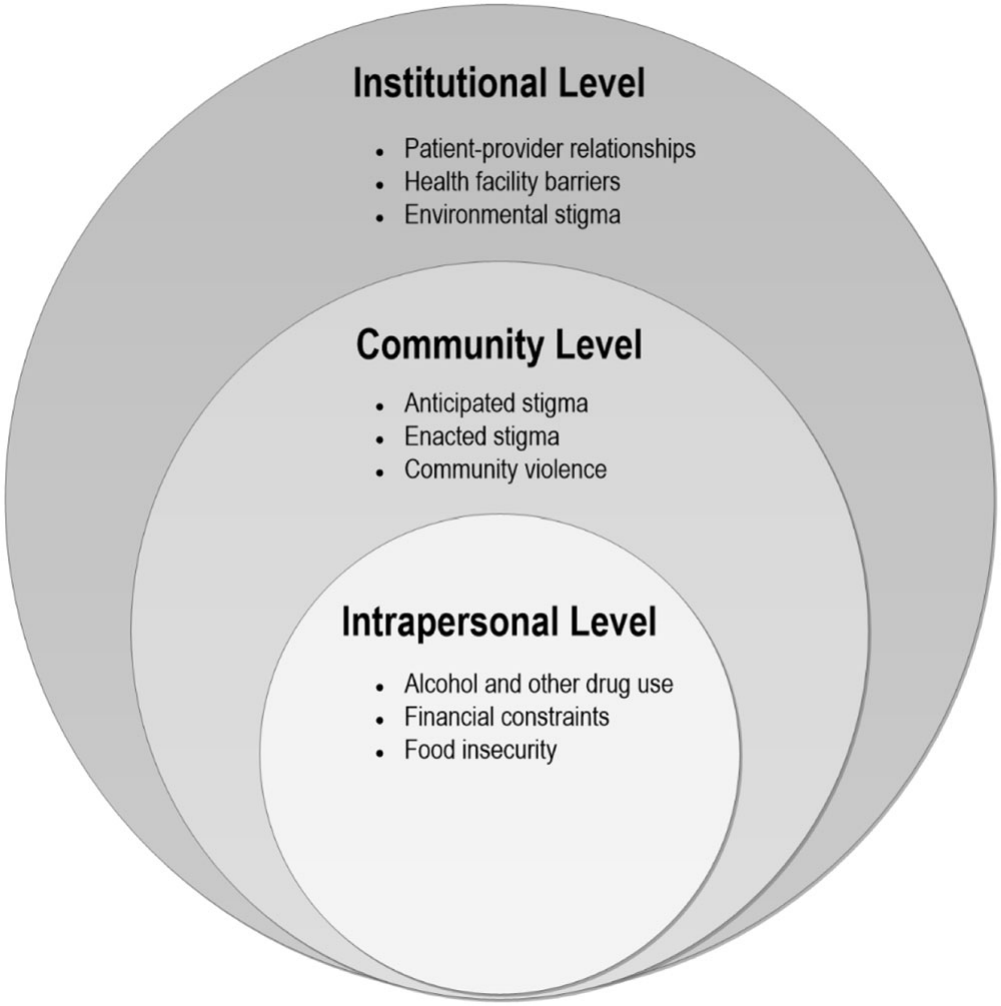
Primary reasons identified for nonadherence to antiretroviral therapy among women living with HIV

The mean age of the participants in this substudy was 33 years (see Table 1). A majority of the women were Black African (94%) and 84% reported having a male partner and an average of two children. Although over two‐thirds of the participants had completed Grade 9 and above, only 5% had completed high school (Grade 12). Approximately 45% of the participants did not have running water in their homes. The mean self‐reported number of drinks per day was 13. The main contextual factors influencing participants’ ART suboptimal adherence found in the analysis are presented under three components of the socioecological framework: intrapersonal level, community level and institutional level (Figure 1).

**Table 1:** Selected participant characteristics

	Total (*N* = 69)
Mean age (years)	33 (SD = 6.0)
Black African	94%
Have a main partner	84%
Number of children (mean)	2 (SD = 1.3)
*Education completed*	
Grades 1–8	29%
Grades 9–11	66%
Grade 12	5%
*Living condition*	
No running water inside the house	45%
House whose walls is made of metallic sheet	45%
Gone to bed hungry at least once in the past year	34%
*Substance use*	
Mean drinks per day in the last 30 days	13 (SD = 7.0)

### 3.1 Intrapersonal level

#### 3.1.1 AOD use

AOD‐use−related barriers were a recurring theme. For some participants, AOD use took precedence over engagement in care. Women reported their struggle with substance use and an inability to attend their clinic appointments to get their medication.

I was drinking too much, hence I decided to stop taking my treatment…. I did not to go back to the clinic because I was not taking the medication…. (Matrix program)

Some women intentionally did not adhere to ART while drinking alcohol because of interactive toxicity beliefs. Participants believed that because both ART and alcohol are drugs, mixing them would lead to adverse health outcomes.

I was told that the ARVs are [a] drug so I did not take medication when I am going to drink alcohol. I would not mix a drug with another drug because I don't know how they are going to affect my body.  (Matrix program)

Toxicity beliefs often stemmed from clinic staff who emphasized alcohol abstinence but concurrently provided conflicting messages on the safety of taking ART while using AODs.

They are confusing us at the clinic because some are saying we must not take our medication if we will be drinking alcohol on that day, then some will say take them before drinking alcohol and wait at least 4 to 5 hours before you start drinking. (HIV/antenatal clinic)

These conflicting messages resulted in women not taking their ART over the weekends when their drinking levels were higher.

#### 3.1.2 Financial constraints

Unemployment and lack of income were reported as a significant challenge to ART adherence. Some participants felt they were not financially stable enough to responsibly commit to taking daily medication and were dependant on their male partners or family for assistance.

I can't depend on someone else for…my treatment. At the end of the day, if I don't eat … I'm gonna get dizzy, or whatever. So that's like starting something that I won't be able to cope with, you understand? But if I had money… then I would say, yeah, I can buy my own groceries every month. No one can tell me I must thank my mother and my father and stay with my fiancé. (HIV/antenatal clinic)

Limited resources led to competing demands. As women who used substances, participants reported they often had to choose between buying substances and providing for other needs, such as food, when they secured some money.

I will just give you an answer. If … you have got those two things [money and HIV], and like you're a user like me. Definitely you know what about choices. Well yes, I'm going to go buy my substance, not food. (Matrix program)

Lack of resources for transportation presented a barrier to health service utilization. Participants expressed difficulty in securing money to pay for public transport to the clinic for their appointment.

It's far [the clinic]. Some of us we are not working. We do not have money for transport [transportation].  (Matrix program)

For some participants who qualified for government financial assistance programmes through disability grants—a lifeline for ART patients that enables them to meet healthcare‐related costs, including transportation, food and treatment access—this assistance was short‐lived as they would lose their grant once their health had rebounded.

And the treatment that we take makes us very hungry and we cannot take treatment on an empty stomach. They refuses to register us for the grants, they will just look at us and assume that we OK or doing good, and we struggling not working. They only give you a grant when you are really sick. (Matrix program)

#### 3.1.3 Food insecurity

Though mentioned sometimes in relation to financial insecurity, food insecurity was another reason for treatment interruption. Participants reported worsening treatment side effects when they took ART without food, leading them to discontinue their medication.

It's about food. (Group ‘Mhm’). ‘Cause if you take that medication and then from that you get sick…it makes you hungry. I don't like to get sick and I don't like to drink that medication. (Matrix program)

Sometimes you don't have food at home…. How can we take care with an empty stomach?  (HIV/antenatal clinic)

Participants reported frustration and helplessness because of a lack of food; consequently, for some, the belief in the inevitability of death because of one's HIV status was adopted as a coping mechanism.

I have defaulted quite a number of times. I sometime[s] think that I am just wasting my time because I am going to die anyway because I am taking the medication on an empty stomach.  (Matrix program)

### 3.2 Community level

#### 3.2.1 Anticipated stigma

Fear of community stigma because of one's HIV‐positive status dissuaded participants from seeking HIV care. Participants reported avoiding going to their clinic for HIV services because they would be recognized by a community member, inadvertently disclosing their HIV status.

Because … sometimes we know each other from the location, and it's obvious when I see [you] here I will conclude that you ‘Nice‐nice’ [living with HIV]. (HIV/antenatal clinic)

Participants shared their concerns about disclosing their HIV status to their partners and family members.

Others can't share with family that they are HIV positive because they are afraid of being discriminated. Others don't share their status with their partners so that makes it difficult for them to take the medication. (HIV/antenatal clinic)

#### 3.2.2 Enacted stigma

Other participants reported personal experiences of the negative consequences of disclosing their HIV status to their family members, which impacted their health‐seeking behaviour.

When they found out that I was HIV positive at home…my own utensils…were always in jik [chlorine bleach] water. They did not even like my child because she was HIV positive and she [was given] to the social workers at the hospital. (HIV/antenatal clinic)

Stigmatizing language and demeaning attitudes held by clinic staff drove away participants from continuing with their healthcare.

They talk to you anyhow, they are sometimes rude to you…it's like when you get infected ne, it's like it was a choice but it's never been a choice. No one goes around looking for HIV. But that is how you are treated at the clinics. (HIV/antenatal clinic)

Participants also reported mistreatment from clinic staff because of missing appointments, which impinged on adherence to medication.

If you have missed your date, they treat you very bad and shout at you. So that makes one feel…scared to go back to the clinic… you end [up] defaulting. (Matrix program)

#### 3.2.3 Community violence

For a few women, fear of violence in the community acted as a physical hindrance to attending the clinic appointment.

So like mine [adherence] has also [gone] down now because for months [I have been] out of my tablets…because…I am in a situation now. Understand? I can't go to the clinic there by me…because…if I'm going to walk on my own because [I might] witnessed a murder. So, I am now home without my tablets. (Matrix program)

Similarly, fear of being robbed or physically harmed for their ART medication forced some women to avoid going to the clinic.

And then when they know what kind of medication you are carrying from the clinic; the gangsters start robbing you by taking your ARV medication by force. (HIV/antenatal clinic)

### 3.3 Institutional level

#### 3.3.1 Patient–provider relationship

Participants reported that clinic staff were dismissive and did not provide adequate information during HIV Counselling and Testing (HCT) concerning their health. They revealed that staff did not show concern nor empathy and they felt unsupported. At times, important HIV knowledge was only imparted after they had defaulted from their treatment. Participants reported feeling disempowered and unable to achieve their health goals.

They test you and tell you that your blood is gone to the lab, when you come back for your date, they just tell you that ‘you OK’. They always say your bloods are ‘OK’, without explaining about some other things. They won't even give a chance to ask more or for any other things, they just say ‘Sisi you OK’, only when you defaulted they send you to the counsellors, you get counselling….  (HIV/antenatal clinic)

Participants reported they were afraid to be honest about their ART adherence because of social desirability that resulted from their clinicians’ zero‐tolerance approach. Some participants reported sharing their missed medication with others to appear to have completed their dose during their follow‐up clinic appointment.

Sometimes you skip a day or 2 not drinking [taking] treatment. So I think you can give out that one you were supposed to take but you skipped… at the clinic when they count it's going to look like you are up to date with your treatment, you have been taking accordingly [laughs]. We all doing it [laughs]. (HIV/antenatal clinic)

Any mechanism for expressing patient satisfaction and redressing corrective action in the clinics, such as suggestion boxes and books, seemed to be defunct. Patients felt discouraged to give feedback as this was often ignored. At times, those who spoke up faced negative consequences, such as being seen last or experiencing rude behaviour.

They are always rushing…. We won't see any change and when you start complaining they will tell you [are] being rude to them, shift your folder for you to be help[ed] last or send you to another sister. The sisters are very rude. (HIV/antenatal clinic)

#### 3.3.2 Health facility barriers

Participants reported they experienced difficulties in refilling their medication when they moved to a new community that was not served by their clinic or if they moved to a new town. The lack of integration of the health facilities' health records frustrated the participants, such as policies where a transfer letter was needed to initiate treatment in a new clinic.

Sometimes you move to another province. When you get there, they will ask for a transfer letter. They can't give you medication if you don't have the transfer letter. (Substance use treatment rehab clinic)

Some clinic administrative barriers hindered women's ability to engage in HIV care. Most clinics did not require appointments. However, women reported that when they went in early to be among the first patients, they could not complete their appointment because of administrative setbacks. One participant described her experience of waiting an entire day to be seen by a provider.

Sometimes I get to the clinic at 7 [in the morning] then I wait until 4 [in the afternoon] and every time I go and ask…they will tell me that my folder is missing, I must sit down and wait, and that's not motivating for me. I would go back home without my medication because of the missing file. (HIV/antenatal clinic)

#### 3.3.3 Environmental stigma

Fear of unintended disclosure was reinforced by various structural characteristics in the clinic environment—such as conspicuously demarcated HIV‐related service departments and different coloured medical records for HIV‐related care—giving a clear indication of what services one was receiving.

[The] thing at the clinic [is] the sections there have different colours on the walls, like people knows if you go into the pink room, you are there for HIV treatment and the yellow room is for something else. (HIV/antenatal clinic)

For some participants, this clinic environment led them to seek care in clinics that were outside of their communities.

## 4 DISCUSSION

The overarching goal of the evidence‐based WHC is to empower women to take better care of their health by reducing alcohol use and adhering to ART. However, although the WHC has been shown to be effective,^22^ there were, in a broader context, barriers to ART adherence and retention in care among women who had completed this intervention. Emerging themes of this substudy revealed these interwoven socioecological factors.

We found that at the individual level, AOD use was the most salient reason for ART interruption. Previous studies have found that individuals who used AODs were more likely to have low adherence 6 months following ART initiation^25^ where the comorbidity of substance use disorders reduces self‐efficacy and self‐care, leading people living with HIV to disengage in healthcare.^26^ Additionally, interactive toxicity concerns between substance use and ART medication have been shown to adversely impact ART adherence.^27^, ^28^ These toxicity concerns often lead to intentional nonadherence or treatment interruptions where people living with HIV stop taking their ART during certain periods when they are using AODs, compromising optimal ART adherence and increasing the likelihood of medication drug resistance. Healthcare providers often discourage substance use while on ART, stressing that substance use undermines the effect of ART.^29^ However, research suggests that the pharmacological effectiveness of ART is not diminished by substance use.^30^ Consequently, educating providers on the minimal impact of substance use on ART efficacy may decrease ART toxicity beliefs and increase ART adherence among those who use substances. Providing further resources for substance use counselling and treatment also may be more effective in increasing ART adherence than exclusively focusing on abstinence.^22^, ^31^, ^32^

It also is well documented that the HIV disease burden among those of lower socioeconomic status is disproportionally high.^33^, ^34^ These findings indicate that lack of financial resources caused by poverty and unemployment negatively affect participants’ ability to engage in HIV care. First, although participants wanted to initiate and remain on treatment, ART became an added obligation where financial constraints already existed. Competing priorities, particularly in low‐resource settings where financial insecurity presents numerous challenges, may lead to nonadherence.^18^, ^33^, ^35^, ^36^, ^37^, ^38^ Second, although ART is free in public health clinics in South Africa, the financial costs of reaching the clinic and lack of childcare and acquiring food to take with medication made daily adherence remain an ongoing issue. These findings are consistent with previous studies that demonstrate that transportation affordability and food insecurity may inadvertently lead to disengagement in care and poor viral suppression.^39^, ^40^, ^41^ To address this, integrated interventions with transport reimbursement^42^ and decentralization of clinic services into community‐based services^43^ have been successful in improving HIV programme outcomes. Providing food supplements and nutritional support to ART patients as a public health service also may improve adherence.^44^, ^45^, ^46^

Government disability social grants provide financial assistance to persons with chronic illnesses such as HIV. However, with eligibility requirements of poor HIV indicators, such as high viral loads, individuals may feel disincentivized to improve their health with the fear of losing their only source of income.^47^ The termination of disability grants when HIV indicators improve should be reviewed and, at the very least, transitioning of individuals into a nutritional and/or transport reimbursement programme may be beneficial.

Women did not feel safe to travel to and from the clinic because of gang violence and increased criminal activity related to having their ART medication stolen. Several studies in South Africa have characterized the use of HIV medication with illicit substances, raising concerns that antiretroviral abuse jeopardizes the safety of both patients and drug users.^48^, ^49^, ^50^ The emerging recreational use of ART, diversion of ART to others through violence and the associated risks of antiretroviral resistance and violence should be at the forefront of community health policy concerns.

Stigma also was attributed to disengagement in care. People living with HIV who reported high levels of stigma are over four times more likely to report poor access to care.^51^ Additionally, HIV‐related stigma has downstream effects on HIV morbidity, mortality and quality of life for people living with HIV.^21^, ^52^ Fear of unintended HIV status disclosure and its repercussion when attending clinic appointments deters health‐seeking behaviour. Often people living with HIV fear attending local community healthcare facilities and will sometimes choose to attend clinics that are far from their local facilities to avoid being recognized.^53^, ^54^ Also, harsh treatment from clinic staff and stigmatizing language often lead to a feeling of moral failing and shame, resulting in treatment interruption. Incorporating facility‐wide stigma‐reduction strategies in staff training and health programmes would foster improved patient–clinic staff relationships.^55^, ^56^ Experiences of stigma may lead people living with HIV to be too afraid to receive much‐needed support from their family members. Normative community stigmatizing beliefs and attitudes related to HIV (community‐level HIV stigma) can negatively impact health‐seeking behaviour among people living with HIV.^57^ Responding to stigma at multiple levels is critical to achieving global targets for HIV testing, linkage to care and viral suppression.^1^

Health service structural barriers, such as health system logistical issues and lack of synergy and integration of health services, increase long wait times, cause delays and have been found to reduce clinic attendance.^58^ Staffing and time constraints may lead to inadequate counselling and lack of rapport building with individuals as they initiate ART.^59^ Healthcare staff also have reported that HIV infection is so prevalent in South Africa that at times it is normalized, desensitizing staff to the extent of education needed by newly diagnosed individuals.^18^

These findings point to the overlapping socioecological barriers related to the treatment journey among women living with HIV who use AODs. It is important to note that despite the barriers to adherence mentioned, all participants reported that being part of the WHC facilitated their attempts to adhere to their medication through social support for AOD use and ART adherence and encouragement from fellow participants.^22^ However, in order for this successful gender‐focused intervention to sustain positive outcomes, it should be coupled with interventions that address broader contextual factors that influence retention in HIV care across multiple domains of women living with HIV with comorbidities such as substance use.

### 4.1 Limitations and strengths

Participants' perspectives and experiences may have been restricted to recall bias. To reduce the likelihood of this bias, focus group discussions were conducted by staff who did not conduct the WHC workshops. The back‐and‐forth translations during the focus groups may have affected the validity of the focus groups’ data. However, these issues were addressed with real‐time interpreters who understood the questions, the social context, cultural background and language spoken by the participants. However, the strengths of this study include the inclusion of a key population that is difficult to reach and is likely to disengage in HIV care. Additionally, the study findings provide validation with qualitative data from healthcare providers regarding barriers to ART adherence among this same population of women living with HIV.^60^

## 5 CONCLUSION

The perspectives of the study participants provided meaningful insight into the external barriers to care among women who had successfully completed a behaviour change intervention emphasizing the importance of understanding the context in which a behaviour takes place. The study findings reflect the broad effects of socioecological barriers to ART adherence that should be addressed in tandem. Foremost, healthcare providers are in a unique position to create a person‐centred experience where ART fits in a patient's complex environment. With the rollout of the universal test‐and‐treat approach, there is a need for further assessment of an individual's ability to initiate and adhere to ART based on underlying challenges, such as AOD use and other social issues. Additionally, continuous booster sessions of brief, effective, gender‐focused interventions, such as the WHC, including screening, counselling and referrals for AOD use integrated into HIV care, may result in longer‐term improvements in ART adherence. Furthermore, as with the WHC, it may be beneficial to integrate community‐based, gender‐focused, support groups and other forms of differentiated care models that provide a safe nondiscriminatory space for patients who have disengaged in care. The goal of these groups would be to provide gender‐specific support in addressing and overcoming barriers to ART adherence; and when stable, individuals could graduate to the conventional ART adherence club or remain to support their peers. Social determinants of health, such as economic stability, affordable housing and transportation, food security and healthcare access have a significant influence on health outcomes. With ART being a lifelong healthcare commitment, comprehensive health services support along the course of the HIV care continuum and government policies that improve the living conditions of people living with HIV will be essential to achieving the 95‐95‐95 UNAIDS targets by 2030. Finally, these recommendations cannot be successful without addressing the drivers and facilitators of multiple‐level stigma, a major barrier to health‐seeking behaviour.

## CONFLICT OF INTERESTS

The authors declare that there are no conflict of interests.

## AUTHORS CONTRIBUTIONS

Jacqueline W. Ndirangu conducted the interviews, interpreted the data, drafted and finalized the manuscript. Wendee M. Wechsberg conceived and designed the study and finalized the manuscript. Margaret W. Gichane and Erin N. Cox coded and analysed the qualitative data. Felicia A. Browne, Courtney P. Bonner, William A. Zule, critically reviewed and revised the manuscript. Kevin M. Smith contributed to the Background section of the manuscript. Tara Carney assisted in conducting the interviews. All authors reviewed, read and approved the final manuscript.

## Data Availability

The data that support the findings of this study are available on request from the corresponding author. The data are not publicly available because of privacy or ethical restriction.
